# Deep-rooted perennial crops differ in capacity to stabilize C inputs in deep soil layers

**DOI:** 10.1038/s41598-022-09737-1

**Published:** 2022-04-08

**Authors:** Leanne Peixoto, Jørgen E. Olesen, Lars Elsgaard, Kirsten Lønne Enggrob, Callum C. Banfield, Michaela A. Dippold, Mette Haubjerg Nicolaisen, Frederik Bak, Huadong Zang, Dorte Bodin Dresbøll, Kristian Thorup-Kristensen, Jim Rasmussen

**Affiliations:** 1 Department of Agroecology, Aarhus University, Blichers Allé 20, 8830 Tjele, Denmark; 2 Division Biogeochemistry of Agroecosystems, University of Goettingen, Buesgenweg 2, 37077 Goettingen, Germany; 3 Department of Plant and Environmental Sciences, University of Copenhagen, Thorvaldsensvej 40, 1871 Frederiksberg C, Denmark; 4 College of Agronomy and Biotechnology/Key Laboratory of Farming System of Ministry of Agriculture and Rural Affairs, China Agricultural University, Beijing, 100193 People’s Republic of China; 5 iCLIMATE Interdisciplinary Centre for Climate Change, Aarhus University, Frederiksborgvej 399, 4000 Roskilde, Denmark; 6 Geo-Biosphere Interactions, University of Tuebingen, Tuebingen, Germany

**Keywords:** Biogeochemistry, Climate change, Agroecology, Climate-change ecology, Microbial ecology

## Abstract

Comprehensive climate change mitigation necessitates soil carbon (C) storage in cultivated terrestrial ecosystems. Deep-rooted perennial crops may help to turn agricultural soils into efficient C sinks, especially in deeper soil layers. Here, we compared C allocation and potential stabilization to 150 cm depth from two functionally distinct deep-rooted perennials, i.e., lucerne (*Medicago sativa* L.) and intermediate wheatgrass (kernza; *Thinopyrum intermedium*), representing legume and non-legume crops, respectively. Belowground C input and stabilization was decoupled from nitrogen (N) fertilizer rate in kernza (100 and 200 kg mineral N ha^−1^), with no direct link between increasing mineral N fertilization, rhizodeposited C, and microbial C stabilization. Further, both crops displayed a high ability to bring C to deeper soil layers and remarkably, the N_2_-fixing lucerne showed greater potential to induce microbial C stabilization than the non-legume kernza. Lucerne stimulated greater microbial biomass and abundance of N cycling genes in rhizosphere soil, likely linked to greater amino acid rhizodeposition, hence underlining the importance of coupled C and N for microbial C stabilization efficiency. Inclusion of legumes in perennial cropping systems is not only key for improved productivity at low fertilizer N inputs, but also appears critical for enhancing soil C stabilization, in particular in N limited deep subsoils.

## Introduction

Deep-rooted agricultural crops, with significant root activity below 1 m, hold the potential to enhance subsoil carbon (C) storage, thereby offsetting anthropogenic CO_2_ emissions^1–4^. However, the strength of this sink remains uncertain due to a lack of empirical data and mechanistic insight in subsoil C deposition. Subsoils are considered to store deposited C more permanently than topsoils, due to the physical disconnect between the soil organic carbon (SOC) and the decomposing heterotrophic microorganisms, coupled with abiotic and biotic subsoil conditions hindering microbial activity^5–10^.

Carbon derived from living roots (i.e., rhizodeposition) ranges from low molecular weight root exudates (i.e., sugars, organic acids, and amino acids) to sloughed off root cells, mucilage, and root hairs^11–13^ and represent the most important C sources for stabilization of SOC in subsoils. The mechanism of subsoil C stabilization is deduced from our current understanding of C turnover in topsoil, where labile C sources are preferentially stabilized in the soil as microbial anabolic remains, which in the form of necromass are integrated in mineral-associated organic matter with mean residence times spanning decades to centuries^9,14–18^. Root exudates, which are labile C sources, and both rapidly and efficiently utilized by a plethora of rhizosphere microbes, display the potential to stimulate subsoil buildup of microbial biomass and subsequent necromass formation through in vivo microbial turnover or anabolic transformations leading to C stabilization^16,19–21^. However, recent studies have shown that subsoil rhizosphere microbes are largely N limited^22–25^, which due to stoichiometric constraints limits the transformation and anabolic stabilization of subsoil C. Removing microbial subsoil N limitations could facilitate in vivo microbial turnover and stabilization of rhizodeposited C in subsoils, but so far it remains elusive if this theoretical target could be operationally achieved, e.g., by additional N inputs from fertilizers or via leguminous N_2_ fixation.

Here, we compared how C rhizodeposition and subsequent microbial stabilization in subsoils to 150 cm was affected by two mineral N fertilizer rates and two functionally distinct deep-rooted perennials: the forage legume lucerne (*Medicago sativa* L.; Family: Fabaceae) and the non-legume kernza, an intermediate wheatgrass (*Thinopyrum intermedium;* Family: Poaceae). Multiple-pulse labeling with ^13^CO_2_ and ^14^CO_2_ was applied to quantify the net rhizodeposited C in field plots of unfertilized lucerne and kernza at fertilizer rates of 100 and 200 kg N ha^−1^ (i.e., K_100_ and K_200_). The relative microbial stabilization of rhizodeposited C was analyzed at depths of 0–25, 25–50, 50–100, and 100–150 cm using an index derived from microbial cell membrane and wall components (phospholipid fatty acids (PLFA) and amino sugars) in conjunction with compound-specific stable isotope probing^26^. Furthermore, amino acids and ^13^C incorporation in roots and rhizosphere soil were analyzed to determine root exudate quantity and quality^27^, and changes in rhizosphere microbial communities and abundance of N cycling genes were assessed.

## Results and discussion

### Belowground C input is decoupled from N fertilizer rate

**Figure 1:**
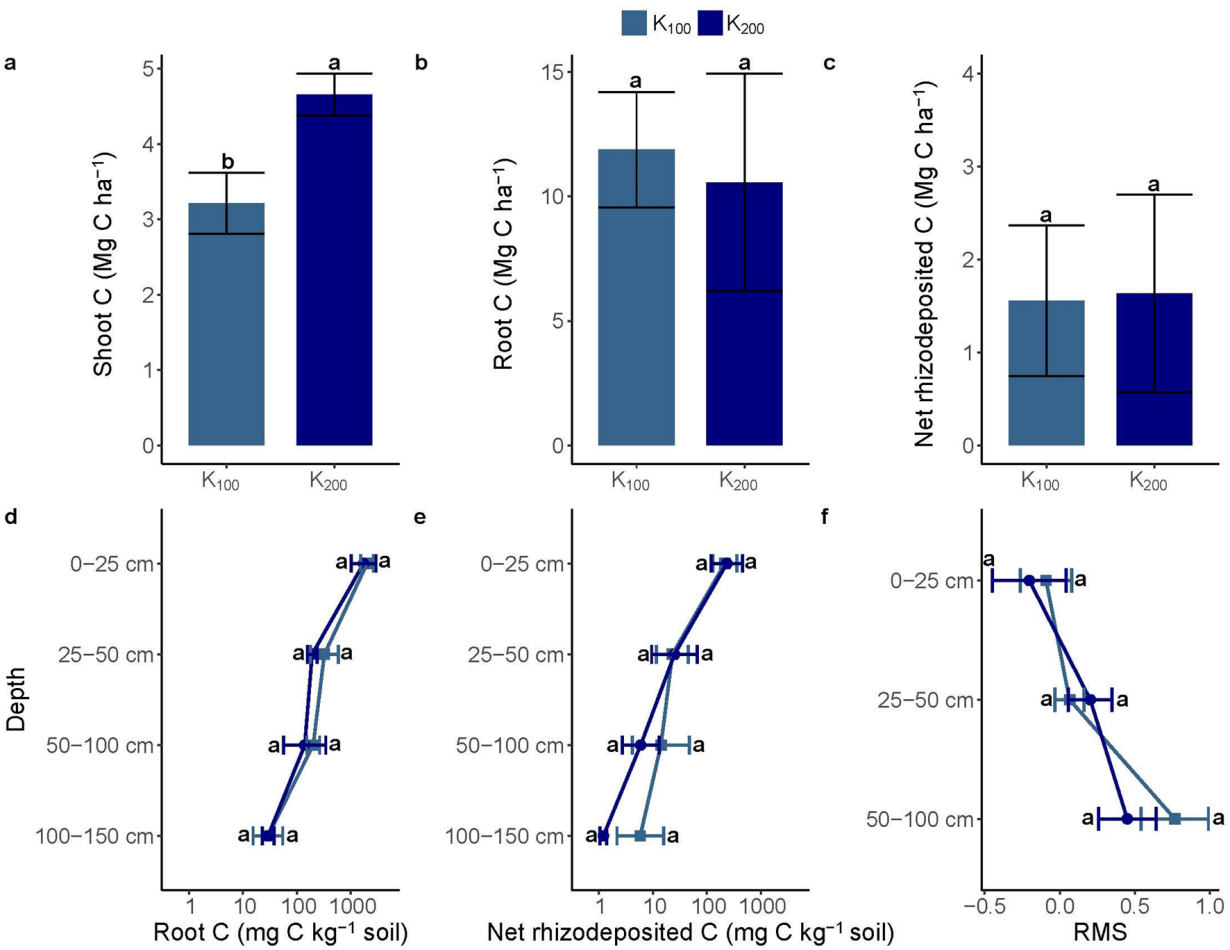
Shoot and root carbon and relative microbial carbon stabilization between kernza fertilized at 100 kg N ha^−1^ (K_100_) and 200 kg N ha^−1^ (K_200_). Shoot C (**a**), total root C (**b**), total net rhizodeposited C (**c**), root C between 0 and 150 cm (**d**), net rhizodeposited C between 0 and 150 cm (**e**), and the relative microbial stabilization (RMS) between 0 and 100 cm (**f**). Statistically significant differences (*p* < 0.05) between K_100_ and K_200_ are indicated by different letters and between K_100_ and K_200_ for each depth of root C and net rhizodeposited C (0–150 cm), and the relative microbial stabilization (0–100 cm). Error bars represent the standard error of the mean, and relative standard errors are shown on the logarithmic plots with a non-log transformed x-axis (**d**,**e**).

Increasing the N fertilization rate from 100 to 200 kg N ha^−1^ increased the aboveground kernza biomass from 7.4 to 10.5 Mg dry matter (DM) ha^−1^, while shoot C increased from 3.2 to 4.7 Mg C ha^−1^ and shoot N from 86 to 184 kg N ha^−1^ (Fig. 1, and Supplementary Fig. S1). However, there was no effect of N fertilizer rate on belowground C input, measured as root C and net rhizodeposited C across depths or at discrete depth intervals (Fig. 1). Likewise, there was no difference in the relative microbial stabilization across all depths (*p* > 0.05) signifying that N fertilizer rate did not affect the microbial stability of top and subsoil rhizodeposited C (Fig. 1). Indeed, the belowground properties of kernza grown with 100 and 200 kg N ha^−1^ were virtually indistinguishable, also for soil and root C and N, the three biomarkers (PLFA, amino sugars, and amino acids), microbial community composition, and associated N cycling gene abundancies (Supplementary Tables S1 and Fig. S2). For these reasons, the two fertilizer treatments were subsequently merged for the statistical analyses of belowground effects of kernza vs. lucerne.

It is well established that higher plant N uptake increases photosynthetic capacity, net primary productivity, and growth of aboveground biomass^28,29^, in line with our results. However, it is also recognized that root-to-shoot ratios are not constant and root inputs to the soil may be decoupled from N fertilizer rate^30^. Additionally, studies by Pausch and Kuzyakov^31^ indicated that the subsoil allocation of newly assimilated C from perennial ryegrass (*Lolium perenne*) was negatively correlated with increasing mineral N fertilization. These observations challenge the notion that increasing N fertilization can potentially increase C deposition in agricultural subsoils^32–36^. In short, a direct link between increasing mineral N fertilization and rhizodeposited C from perennial deep-rooted grass crops is not likely. However, exploring the effects of fertilization at the lower end of the fertilization rates would be of relevance for exploring long-term effects of perennial cropping systems.

### Deep-rooted legumes improve subsoil C input and stabilization

**Figure 2:**
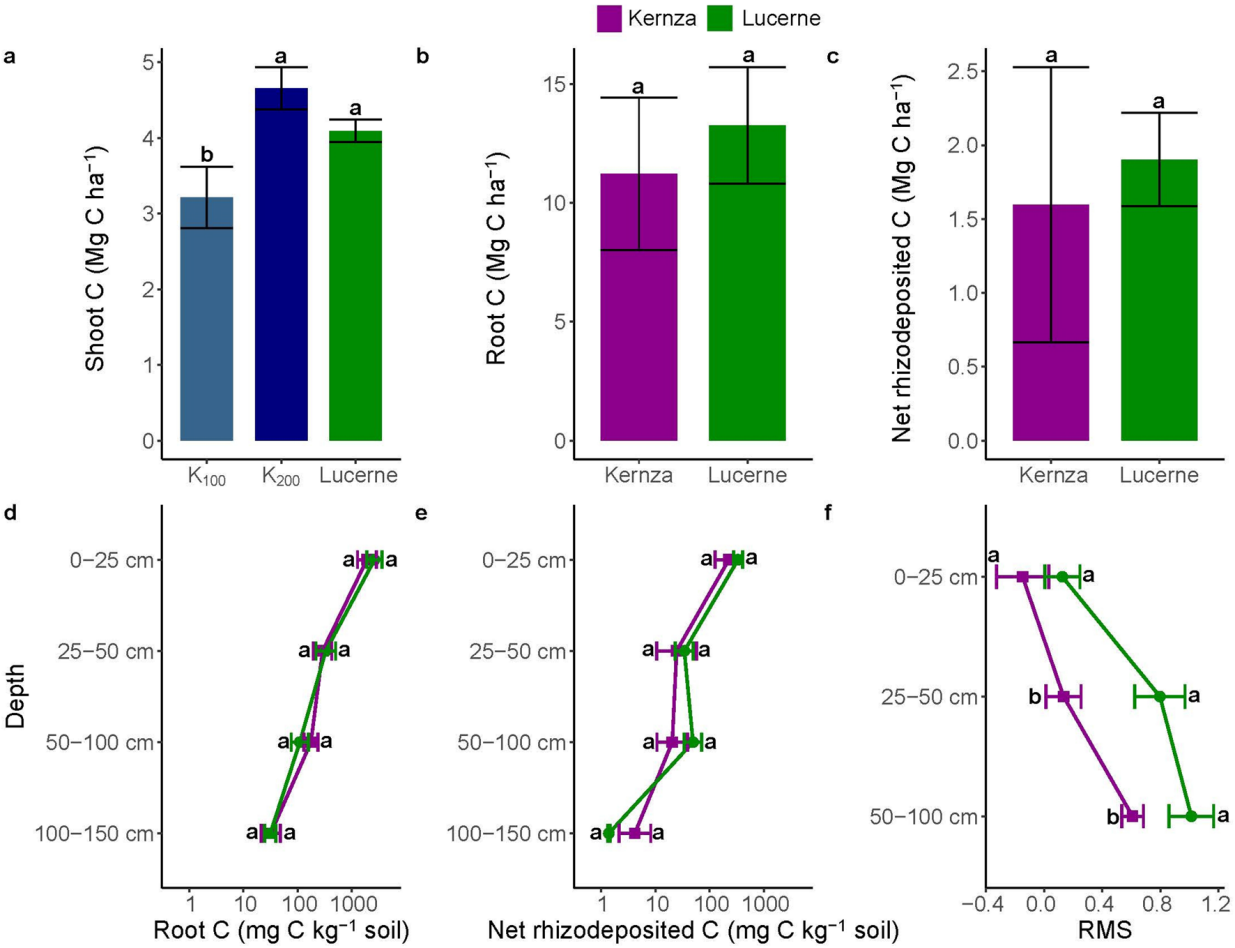
Shoot and root carbon and relative microbial carbon stabilization between kernza and lucerne. Shoot C between kernza at both 100 and 200 kg N ha^−1^ and lucerne (**a**), total root C (**b**), total net rhizodeposited C (**c**), root C between 0 and 150 cm (**d**), net rhizodeposited C between 0 and 150 cm (**e**), and the relative microbial stabilization (RMS) between 0 and 100 cm (**f**). Statistically significant differences (*p* < 0.05) between kernza and lucerne are indicated by different letters and between plant species for each depth of root C and net rhizodeposited C (0–150 cm), and the relative microbial stabilization (0–100 cm). Error bars represent the standard error of the mean, and relative standard errors are shown on the logarithmic plots with a non-log transformed x-axis (**d**,**e**). The two fertilizer application rates of kernza were merged due to non-significant differences and collectively referred to as kernza (**b**–**f**).

The unfertilized lucerne had an aboveground biomass production comparable to kernza at the fertilization rate of 200 kg N ha^−1^ (Supplementary Fig. S1), whereas the aboveground biomass and shoot C of K_100_ were one- to twofold lower than lucerne (Fig. 2 and Supplementary Fig. S1). There was no effect of plant species on total root C (11.2–13.3 Mg C ha^−1^) and net rhizodeposited C (1.6–1.9 Mg C ha^−1^), nor did these C inputs differ between plant species within each depth interval down to 150 cm (Fig. 2). Importantly, between 25 and 100 cm, the relative microbial C stabilization was higher for lucerne than kernza (*p* = 0.27 for 0–25 cm, 0.01 for 25–50 cm, and 0.03 for 50–100 cm), implying a significantly greater microbial stabilization of rhizodeposited C derived from lucerne.

Over the entire depth range, there were no notable differences in recovered roots (Supplementary Table S2). However, lucerne exhibited a higher root N content, markedly lower root C:N ratio across all depths, and higher rhizodeposited N between 50 and 100 cm than kernza (Supplementary Table S2). At the biomarker level, there were minimal differences in total amino sugars, ^13^C amino sugars, and ^13^C PLFA in the rhizosphere soil between lucerne and kernza (Supplementary Table S2). Yet, the total PLFA was generally higher for lucerne than kernza, implying a greater living microbial biomass within the lucerne rhizosphere (Supplementary Table S2). The total amino acids of the roots were higher for lucerne as compared to kernza across all measured depths with a tendency also towards higher total amino acids in the lucerne rhizosphere soil (Supplementary Table S2). Lucerne likewise had a higher total ^13^C content of amino acids in the roots, whereas no clear trends were detected for total ^13^C amino acids in rhizosphere soil between lucerne and kernza (Supplementary Table S2).

At the molecular level, there was an effect of plant species and depth on the bacterial and fungal communities showing divergent microbial communities under lucerne and kernza that become more dissimilar with depth (Supplementary Fig. S2). In addition, the relative abundance of unique bacterial and fungal amplicon sequencing variants (ASVs) increased with depth, suggesting that the deep rhizosphere communities are more plant specific than the top communities (Supplementary Table S3). Thus, lucerne and kernza harbor distinct microbial communities that become more dissimilar with depth when C and N become increasingly limited^24^, suggesting a plant-mediated effect on the colonization or recruitment of these deep soil rhizosphere microbes^11,37^. Further, the abundance of the nitrogenase gene (*nifH*), involved in N_2_-fixation, was generally highest under lucerne, but lucerne subsoils also showed an overall higher abundance of nitrite reductase (*nirK, nirS*), nitrous oxide reductase (*nosZ*), and ammonia monooxygenase (*amoA*) genes (Supplementary Table S2), suggesting a more dynamic microbial N cycling in subsoils under lucerne than kernza.

Kernza and lucerne both displayed the capacity for C allocation to C-depleted subsoils via root tissue and rhizodeposition. So far, however, most studies on belowground C rhizodeposition have been restricted to topsoils. Of the few studies comprising subsoils, the net C input (i.e., root biomass and rhizodeposition) from lucerne grown for 150 days was 3.9 Mg C ha^−1^ in the topsoil (0–30 cm) and 3.8 Mg C ha^−1^ between 30 and 105 cm, which was eightfold higher in the subsoil as compared to deep-rooted chicory (*Cichorium intybus* L.)^38^. These values are exceedingly lower as compared to the present net C input from lucerne in the topsoil (11.5 Mg C ha^−1^ at 0–25 cm), but in agreement with the net C input between 25 and 150 cm (3.6 Mg C ha^−1^). This is likely due to different growth periods and site-specific soil conditions. Both Peixoto, et al.^26^ and the present study showed an increased relative microbial stabilization with depth suggesting a dominance of C transformed into microbial necromass and thus a high potential for C stabilization in deeper soils.

### Quality matters: coupled C and N dynamics drives microbial stabilization

**Figure 3:**
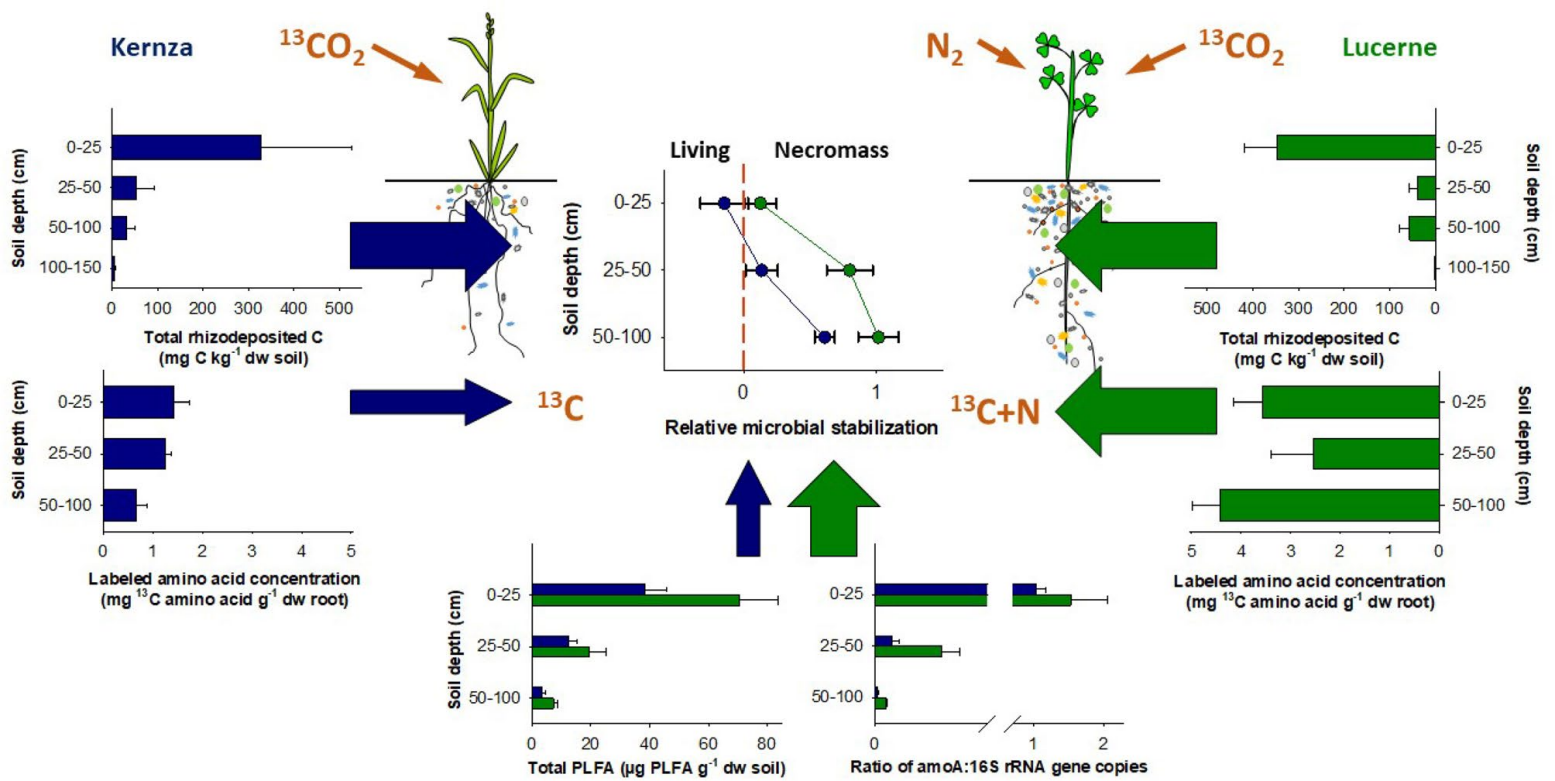
The variables related to the higher relative microbial stabilization under lucerne (green) than kernza (blue) despite similar total C inputs via rhizodeposition (upper panels). The relative microbial stabilization below zero shows that ^13^C is primarily in the living microbial biomass and a value above zero shows that ^13^C has shifted into the microbial necromass. Lucerne roots have a greater content of labeled amino acids (middle panels) and induce a higher rhizosphere soil amino acid content (Supplemental Table S2) as evident by the greater abundance of N cycling genes in lucerne rhizosphere soil (right lower panel). The greater coupling of C and N within the lucerne rhizosphere along with greater microbial biomass (Total PLFA, lower panel) induced a higher relative microbial stabilization of rhizodeposited C under lucerne.

The greater capacity of lucerne to induce microbial C stabilization is likely linked to plant–microbe-interactions where root and exudate C:N ratio and amino acid content have cascading effects on microbial communities and eventual microbial C stabilization (Fig. 3). The higher root content of N and N-rich amino acids provides a discernable difference in the quality of organic compounds leaving the roots of lucerne as compared to kernza. Consequently, the higher abundance of N cycling genes in the lucerne rhizosphere is linked to the exudation of more N-rich root-derived compounds (e.g., free or peptide/protein-bound amino acids). This effectively transforms the C and N depleted deep-soil environment into an enriched rhizosphere promoting the proliferation of microbes, as seen by the higher living rhizosphere microbial biomass (Supplementary Table S2), thereby coupling C and N assimilation in biomass under lucerne. Thus, a likely scenario is that removing both the C and N microbial limitation^24^ triggered not only a higher living microbial biomass, but also a higher relative microbial stabilization of lucerne-derived C through greater microbial anabolism of organically bound C and N (i.e., amino acids, peptides, and proteins)^39^, where subsequent in vivo microbial turnover^21^ led to necromass formation^16,19,20,40^. Hence, we show that deep-rooted perennial crops display the potential to enhance deep soil C storage, and lucerne displays a greater capacity to induce microbial stabilization. Utilizing the capacity of deep soil for C storage requires further studies building upon our novel findings to resolve the magnitude of subsoil C input and stabilization across a wider range of pedo-climatic conditions and plant species that would allow incorporation into models for upscaling of the potentials.

## Materials and methods

### Experimental design and crop management

The study was conducted during 2019 in a field experiment on an arable soil (classified as Luvisols) in the deep root experimental facility at the University of Copenhagen, Denmark (Supplementary Table S4). The experiment was conducted with two diverse perennial deep-rooted species: the tap-rooted forage legume lucerne (*Medicago sativa* L. (cv. Creno); Family: Fabaceae) with the capacity to fix N_2_ and the intermediate wheatgrass (*Thinopyrum intermedium;* Family: Poaceae) kernza developed by the Land Institute (Salina, Kansas, USA). Kernza was initially sown on April 11th, 2015 and lucerne on September 9th, 2016 with a seeding density of 20 kg seeds ha^−1^. Every year, kernza was fertilized with NPK fertilizer (21:7:3; NH_4_:NO_3_ = 1.28) as a single dose in early spring (before the onset of plant growth). Kernza was harvested every year in August using a combine harvester and lucerne three times per year in June, August, and October. Plants were rainfed with a subsurface drain installed at both 1 and 2 m depth running between the plots.

For each species, fixed frames of 0.75 m^2^ were inserted in the soil (ca. 5 cm) within each field plot. Specifically, three field plots of lucerne (with observable root nodulation) and kernza were used where each of the three kernza field plots contained two subplots of N fertilized kernza at 100 kg N ha^−1^ (K_100_) (i.e., the standard fertilization within this field) and N fertilized kernza at 200 kg N ha^−1^ (K_200_) (i.e., within the range of standard fertilization practices for kernza). Before the onset of plant growth, all plots received ^15^N (as ^15^NH_4_Cl; 98 atom%) in trace amounts (corresponding to 1 kg N ha^−1^) to trace N allocation from the surface to deeper layers.

### ^13^C/^14^C-CO_2_-labeling

Within each fixed frame, the ^13^C/^14^C-CO_2_-labeling was conducted using an atmospheric labeling chamber^41^. Labeling with C-tracers was done with multiple-pulse labeling (three times per week) over two months until first harvest (May 2nd to June 20th 2019). Glass beakers containing ^13^C labeled bicarbonate (0.1 g mL^−1^ labeling solution; 99 atom%), and ^14^C labeled bicarbonate (11 kBq mL^−1^ labeling solution) within a solution of NaOH (1 M) were added within each of the labeling chambers. Once chambers were sealed, hydrochloric acid (HCl; 2 M) was added to the labeling solution (in equivalent amounts) via a syringe promoting ^14^CO_2_/^13^CO_2_ evolution. Chambers remained sealed for one to three hours (between 9 am and 12 pm) depending on weather conditions (i.e., the duration and intensity of sunshine). The amount of added labeling solution sequentially increased with increasing plant growth (i.e., 5 mL per 20 cm increase in plant height) reaching a plant height of 100–120 cm at the termination of the labeling.

### Shoot, root, and soil sampling

The labeling plots (0.75 m^2^) were harvested on June 20th, 2019 to obtain the aboveground biomass of lucerne and kernza (K_100_ and K_200_). The aboveground biomass in addition to samples obtained from unlabeled parts of the field was directly stored at − 20 °C until drying at 105 °C for two days. For each plot and unlabeled samples, the plant biomass was homogenized and ball-milled for subsequent isotopic analyses.

Soil cores to 1.5 m depth were taken inside all labeling plots, and cores were subdivided into four depth intervals: 0–25, 25–50, 50–100, and 100–150 cm. The soil coring was conducted in 25 cm intervals using a soil auger (6 cm inner diameter). Specifically, per depth three soil samples were taken and stored at 4–5 °C (ca. two days) and then immediately processed and stored at -20 °C until analyses. Roots, bulk soil and rhizosphere soil (adhering to the roots), were separated by sequential sieving of the soil with finer mesh sizes to 1 mm as described by Peixoto, et al.^26^. A subsample of the bulk soil (ca. 150 g) from each depth in all labeling plots was washed on a 250 µm sieve to recover root fragments for subsequent isotopic determination in unrecovered root fragments. Soil samples (and associated roots) from unlabeled parts of the larger field plots were used to determine natural abundance of ^13^C/^14^C/^15^N with depth. The collection of plant material complied with relevant institutional guidelines and seeds were gifted by University of Copenhagen.

### Determination of ^13^C/^14^C/^15^N enrichment, and C and N quantity

For each defined depth, samples of roots and soil were homogenized, freeze-dried (except PLFA samples that were stored at − 20 °C), and ground in a ball-mill for the determination of total C and N, ^13^C, ^15^N, and ^14^C activity. Total C, N, ^13^C, and ^15^N were measured with a FLASH 2000 CHNS/O Elemental Analyzer (Thermo Fisher Scientific, Cambridge, UK) combined to a Delta V Advantage isotope ratio mass spectrometer via a ConFlo III interface (Thermo Fisher Scientific, Bremen, Germany) at the Centre for Stable Isotope Research and Analysis (Georg August University Göttingen, Göttingen, Germany).

All δ^13^C values are standardized to the Vienna PeeDee Belemnite international isotope standard and δ^15^N values standardized to the δ^15^N values of atmospheric N_2_. ^13^C and ^15^N enrichment is expressed as atom% excess as calculated by the atom% difference between the respective labeled and unlabeled samples. The ^14^C activity was determined by combustion in a Hidex 600 OX Oxidizer (Hidex, Turku, Finland) and counted on a liquid scintillation counter (Tri-Carb 3180TR/SL, PerkinElmer, Waltham, MA, USA). ^14^C enrichment is determined by the difference in the ^14^C activity (Bq g^−1^) between the respective labeled and unlabeled samples.

### Calculation of root C and net rhizodeposition

The amount of root C (mg C kg^−1^ soil) was calculated based on the root dry matter and C concentration divided by the quantity of soil sampled^38^. For the determination of net rhizodeposition, ^14^C was used due to lower detection limits in deeper soil layers^42^. A modified tracer mass balance approach described by Rasmussen, et al.^43^ with adjusted unrecovered root fragments^41^ was used to determine the net rhizodeposition based on the following equations where the %ClvR is the relative proportion of rhizodeposition expressed as the percent C lost via rhizodeposition:

$${\text{\%ClvR}} = \frac{{^{{{14}}} {\text{C Soil (rhizosphere + adjusted bulk)}}}}{{^{{{14}}} {\text{C bulk soil }} + \,^{{{14}}} {\text{C rhizosphere soil}} + \,^{{{14}}} {\text{C Root}}}} \times 100.$$

$${\text{Net rhizodeposition}} = \frac{{{\text{\%ClvR }} \times {\text{ root C content}}}}{{\left( {100 - \% {\text{ClvR}}} \right)}}$$

The ^14^C soil content was the sum of the adjusted bulk soil ^14^C and rhizosphere ^14^C content for each soil sample. The ^14^C rhizosphere and bulk soil content for each soil sample were determined by multiplying the total quantity of C by the ^14^C enrichment of the soil. The adjusted bulk soil ^14^C content was calculated as the difference between the bulk ^14^C soil content by the ^14^C root washed content as determined by the multiplication of ^14^C enrichment in root fragments recovered from a subsample of soil by the total C content within the entire soil volume sampled. The ^14^C root content was determined by multiplying the total quantify of C in roots by the ^14^C enrichment. Similar equations were used to calculate the net rhizodeposition of N based on ^15^N enrichment within the soil and roots.

### Biomarker analyses

#### Phospholipid fatty acid (PLFA)

The analysis of PLFAs was done according to a modified protocol by Frostegård, et al.^44^ with a detailed description of the modifications provided by Gunina, et al.^45^. In brief, 25 μL of 1,2-Dinonadecanoyl-sn-Glycero-3-Phosphatidylcholine (C19:0) (1 mg mL^–1^) were added to each of the samples and used in the quantification of recovery of the phospholipids. The lipid fraction from 5–6 g of rhizosphere soil was extracted twice using a one-phase Bligh-Dyer extractant^46^ of chloroform, methanol (MeOH), and citrate buffer (pH 4) (1:2:0.8, v/v/v). To isolate the phospholipid fraction, a solid-phase extraction with activated silica gel and methanol elution was conducted. The derivatization into fatty acid methyl esters occurred via a sequential hydrolyzation with 0.5 mL sodium hydroxide (NaOH) (0.5 M) in MeOH for 10 min at 100 °C and methylation with 0.75 mL of boron trifluoride (BF_3_) (1.3 M) in MeOH for 15 min at 80 °C. An external standard stock solution containing 28 individual fatty acids (ca. 1 mg mL^–1^ per fatty acid) used in the quantification of PLFA content was simultaneously derivatized with the samples. The residues were dissolved in 185 μL of toluene, and 15 μL of the internal standard 2, tridecanoic acid methyl ester (C13:0) (1 mg mL^–1^) were added to each sample prior to measurement using an Agilent 7820A GC coupled to an Agilent 5977 quadrupole mass spectrometer (Agilent, Waldbronn, Germany). The sum of all PLFAs was used as a proxy of the living microbial biomass based on the direct relation between PLFAs and microbial biomass.

#### Amino sugars (AS)

Amino sugars were extracted according to a modified protocol by Zhang and Amelung^47^ with a detailed description of the procedure by Peixoto, et al.^26^. In brief, 0.8–1.5 g of freeze-dried rhizosphere soil were hydrolyzed with the addition of 11 mL of 6 M HCl for 8 h at 105 °C. Following hydrolysis, soil samples were filtered and HCl was removed via rotary evaporation at 45 °C to dry the filtrate. Prior to derivatization both iron precipitates and salts were removed from the filtrate and 25 μL of the internal standard 1, methylglucamine (MeGlcN) (1 mg mL^–1^) was added and used for quantification of recovery. The derivatization into aldononitrile acetates was conducted as described by Zhang and Amelung^47^. For the quantification of AS, an external standard stock solution containing the AS: *N*-acetylglucosamine (GlcN) (2 mg mL^–1^), *N*-acetylgalactosamine (GalN) (2 mg mL^–1^), *N*-acetylmuramic acid (MurN) (1 mg mL^–1^), mannosamine (ManN) (2 mg mL^–1^), and MeGlcN (1 mg mL^–1^) was derivatized and analyzed with the samples. The residues were dissolved in 185 μL of ethyl acetate-hexane (1:1, v/v), and 15 μL of the internal standard 2, tridecanoic acid methyl ester (1 mg mL^–1^), were added to the samples for measurement using an Agilent 7890A GC coupled to Agilent 7000A triple quadrupole mass spectrometer (Agilent, Waldbronn, Germany). Total amino sugars content was calculated as the summation of the four detected amino sugars: GlcN, MurN, GalN, and ManN.

#### Amino acids (AA)

Amino acids were extracted from both freeze-dried rhizosphere soil and root samples according to the protocol by Enggrob, et al.^48^. In brief, 0.8–3 g of rhizosphere soil and 0.02 g of root were hydrolyzed with the addition of 2 mL of 6 M HCl for 20 h at 110 °C to break the peptide bonds. Samples were subsequently purified via the removal of lipophilic and solid compounds by the addition of 4 mL *n*-hexane/dichloromethane (6:5, v/v) to the soil and root samples. Following centrifugation, the aqueous phase was filtered through glass wool and rinsed with 2 × 0.5 mL 0.1 M HCl into new glass tubes with the addition of 300 μL of the internal standard, norleucine (2.5 mM). The samples were freeze-dried and the residues dissolved in 1 mL 0.01 M HCl prior to the separation of amino acids and amino sugars (i.e., N containing compounds) on a polypropylene column with a cation exchange resin. The amino acids were eluted with a 2.5 M ammonium hydroxide solution and freeze-dried prior to derivatization of the amino acids as described by Enggrob, et al.^48^. For the quantification of AA, an external standard stock solution containing 14 AA was derivatized and analyzed with the samples. The amino acids were measured using a trace GC Ultra mounted with a TriPlus autosampler (Thermo Scientific, Hvidovre, Denmark) coupled via a combustion reactor (GC IsoLink, Thermo Scientific) to an isotope ratio mass spectrometer (Delta V Plus IRMS, Thermo Scientific). The total AA content of the rhizosphere soil and roots was based on the summation of the AA: alanine, Asx (asparagine and aspartate), Glx (glutamine and glutamate), glycine, isoleucine, lysine, phenylalanine, Pro/Thr (proline and threonine), serine, tyrosine, and valine.

### Compound-specific stable isotope probing

To determine the ^13^C enrichment of biomarkers, all raw δ^13^C were measured individually for AS and PLFA using a Delta V Advantage isotope ratio mass spectrometer via a ConFlo III interface (Thermo Fisher Scientific, Bremen, Germany). For AA, all raw δ^13^C were measured using a trace GC Ultra mounted with a TriPlus autosampler (Thermo Scientific, Hvidovre, Denmark) coupled via a combustion reactor (GC IsoLink, Thermo Scientific) to an isotope ratio mass spectrometer (Delta V Plus IRMS, Thermo Scientific). For each sample, chromatogram peaks identified based on retention times specific for the measured amino sugars, PLFA, and AA were integrated using Isodat v. 3.0 (Thermo Fisher Scientific). All raw δ^13^C values were corrected for dilution by additional C atoms added during the derivatization, amount dependence, offset, and drift (for PLFA samples)^49–51^. To determine the ^13^C incorporation into each biomarker, the ^13^C excess for each biomarker as determined by the difference between the ^13^C of the labeled and unlabeled biomarker was multiplied by the C content of the specific biomarker.

### Relative microbial stabilization (RMS)

The relative microbial stabilization is based on the relation of rhizodeposited ^13^C in the PLFA and amino sugar pools as described in detail by Peixoto, et al.^26^. The underlying assumption is that ^13^C incorporation into the amino sugar pool indicates the transformation of rhizodeposited C into necromass^52,53^, and the ^13^C incorporation into the PLFA pool (i.e., the living microbial biomass) represents a temporary C pool as PLFAs are immediately exposed to degradation following cell lysis^54^. The relative microbial stabilization (RMS) is calculated as follows:

$${\text{Relative microbial stabilization}} = {\text{log}}\frac{{{\text{Average weighted atom\% }}\,^{{{13}}} {\text{C excess AS}}}}{{{\text{Average weighted atom\% }}\,^{{{13}}} {\text{C excess PLFA}}}}$$

where the average weighted atom% ^13^C excess is determined by the total ^13^C incorporation divided by the total C content of the respective PLFA or amino sugar pools. Accordingly RMS < 0 reflects C allocated into living microbial biomass being a short-lived C pool, whereas RMS > 0 is indicative of higher stabilization of C based on the dominant entry of C into the microbial necromass. However, the RMS indicator does not imply the absolute stability of rhizodeposited C, but rather signifies the potential for microbial stabilization among contrasting experimental variables (i.e., depth and plant species).

### Molecular analysis

#### DNA extraction

From each sample, 0.5 g of freeze-dried rhizosphere soil was used for DNA extraction using the Fast DNA Spin kit for Soil (MP Biomedicals, Solon, OH, USA) according to the manufacturer’s protocol with a single modification. Following, the addition of Binding Matrix, the suspension was washed with 5.5 M Guanidine Thiocyanate (protocol from MP Biomedicals) to remove humic acids that could inhibit preceding polymerase chain reaction (PCR) steps. The DNA was eluted in DNase free water and purified using the NucleoSpin gDNA Clean-up kit following the manufacturer’s protocol (Macherey–Nagel, Düren, Germany). The purity and concentration of DNA were checked on Nanodrop and Qubit, respectively.

#### Amplicon sequencing

Extracted DNA was sent to Novogene Europe (Cambridge, United Kingdom) for library preparation and amplicon sequencing. For 16S rRNA gene amplicon sequencing of the V3-V4 regions, the primer pair 341 F and 806 R were used (Supplementary Table S5). To identify the fungal communities, we targeted the Internal Transcribed Spacer (ITS) Region 1, using the primer pair ITS1 and ITS2 (Supplementary Table S5). The constructed libraries were sequenced using a Novaseq 6000 platform producing 2 × 250 bp paired-end reads. Raw sequences were deposited in the NCBI Sequence Read Archive (Bioproject number PRJNA736561).

#### Quantitative PCR

Copy numbers of the 16S rRNA gene were determined by quantitative PCR (qPCR) using the primers 341F and 805R (Supplementary Table S5) on an AriaMX Real-Time PCR System (Agilent Technologies, Santa Clara, CA, USA). An external plasmid standard curve was made based on the pCR 2.1 TOPO vector (Thermo Fisher Scientific, Waltham, MA, USA) with a 16S rRNA gene insert amplified from bulk soil. The PCR reaction was performed in 20 µl reactions containing: 1 × Brilliant III Ultra-Fast SYBR green low ROX qPCR Master Mix (Agilent Technologies, Santa Clara, CA, USA), 0.05 µg/µl BSA (New England Biolabs Inc., Ipswich, MA, USA), 0.4 µM of each primer and 2 μl of template DNA. The thermal cycling conditions were 3 min at 95 °C followed by 40 cycles of 20 s at 95 °C and 30 s at 58 °C, and a final extension for 1 min at 95 °C. A melting curve was included according to the default settings of the AriaMx qPCR software (Agilent Technologies). The reaction efficiencies were between 97 and 102%. Fungal quantification was done by qPCR amplification of the Internal Transcribed Spacer 1 (ITS1) using the primers ITS1-F and ITS2 (Supplementary Table S5). A plasmid standard curve was made using the pCR 2.1 TOPO vector containing an ITS1 region from *Penicillium aculeatum*. Reaction mixture and cycling conditions were as described above for the 16S rRNA gene (Supplementary Table S5). The reaction efficiency was 84%.

#### Quantification of functional genes involved in N cycling

The five bacterial genes *amoA*, *nirK*, *nirS*, *nosZ*, and *nifH* coding for enzymes involved in N-cycling were quantified by qPCR on an AriaMx Real-Time PCR System (Agilent Technologies). Reaction mixtures and cycling conditions were as described above for the 16S rRNA gene (Supplementary Table S5). The standard curves were prepared as described in Garcia-Lemos, et al.^55^. The reaction efficiencies were in the range 87%-105%.

#### Sequence processing

Raw reads were treated using DADA2 version 1.14.1^56^. In brief, reads were quality checked and primers were removed using Cutadapt v. 1.15^57^. We followed the protocol DADA2 using default parameters, with a few modifications. For 16S rRNA sequences, the forward and reverse reads were trimmed to 222 and 219 bp, respectively, while the maxEE was set to 2 and 5 for forward and reverse reads, respectively. Detection of amplicon sequence variants (ASVs) was done using the pseudo-pool option and forward and reverse reads were merged with a minimum overlap of 10 bp. Merged reads in the range of 395–439 bp were kept, as reads outside this range are considered too long or too short for the sequenced region. Taxonomy was assigned using the Ribosomal Database Project (RDP) classifier^58^ with the Silva database v.138^59^. For ITS region 1, quality filtered reads shorter than 50 bp were removed prior to merging the forward and the reverse reads, with maxEE set to two for both forward and reverse reads. During merging, the minimum overlap was set to 20 (default). Taxonomy was assigned with the RDP classifier using the Unite v. 8.2 database^60^ after removal of chimeras.

As ITS region 1 has a variable length, reads can be lost during merging. Hence, to validate our dataset we ran only the forward reads through the DADA2 pipeline and compared the overall community structure with the dataset from the merging using a Mantel test. No significant changes were observed in the community structures between the two datasets (*r* = 0.99; *p* = 0.0001). To obtain the highest taxonomic resolution, the dataset based on the merged reads was used. Further analysis was done using the phyloseq v. 1.30.0 R package^61^.

### Statistical analysis

Analyses of variance (ANOVA) were conducted to examine the effects of N fertilized kernza at 100 kg N ha^−1^ (K_100_) and kernza at 200 kg N ha^−1^ (K_200_) as well as to test the effect of the deep-rooted plant species: kernza and lucerne, and soil depth on each of the dependent variables. An average across the two subplots within each of the three kernza field plots was used when measured variables did not significantly differ between K_100_ and K_200_. Subsequent pairwise comparisons of the means was conducted using the TukeyHSD post-hoc test. Homogeneity of variance and normality were confirmed (data log-transformed when required) for all comparisons using the Fligner-Killeen test of homogeneity of variances^62^ and the Shapiro–Wilk test of normality^63^. A permutational multivariate analysis of variance (PERMANOVA) using the Bray–Curtis dissimilarity matrix with the adonis function in the vegan R package was used to test the effect of K_100_ and K_200_, lucerne across both K_100_ and K_200_, and depth on the bacterial and fungal communities. The multivariate homogeneity of group dispersions or variances were confirmed for all comparisons using the function betadisper in vegan. The bacterial and fungal communities in response to the ascribed variables were visually represented as ordination plots with a Principle Coordinates Analysis (PCoA). Unique ASVs were defined for each depth and between K_100_, K_200_, and lucerne as ASVs only present in those samples belonging to a specific depth and treatment. Significance testing was conducted at *p* < 0.05. All statistical analyses and graphics were conducted in R^64^.

## Supplementary Information

Supplementary Information.

## Data Availability

Data available on request from the authors.

## References

[1] Kell DB (2012). Large-scale sequestration of atmospheric carbon via plant roots in natural and agricultural ecosystems: Why and how. Philos. Trans. R Soc. Lond. B Biol. Sci..

[2] Kell DB (2011). Breeding crop plants with deep roots: Their role in sustainable carbon, nutrient and water sequestration. Ann Bot.

[3] Pierret A (2016). Understanding deep roots and their functions in ecosystems: An advocacy for more unconventional research. Ann. Bot..

[4] Thorup-Kristensen K (2020). Digging deeper for agricultural resources, the value of deep rooting. Trends Plant Sci..

[5] Salomé CM, Nunan N, Pouteau VR, Lerch TZ, Chenu C (2010). Carbon dynamics in topsoil and in subsoil may be controlled by different regulatory mechanisms. Glob. Change Biol..

[6] Sanaullah M (2011). Decomposition and stabilization of root litter in top- and subsoil horizons: what is the difference?. Plant Soil.

[7] Pries CEH (2018). Root litter decomposition slows with soil depth. Soil Biol. Biochem..

[8] Gleixner G (2013). Soil organic matter dynamics: a biological perspective derived from the use of compound-specific isotopes studies. Ecol. Res..

[9] Cotrufo MF, Ranalli MG, Haddix ML, Six J, Lugato E (2019). Soil carbon storage informed by particulate and mineral-associated organic matter. Nat. Geosci..

[10] Schmidt MW (2011). Persistence of soil organic matter as an ecosystem property. Nature.

[11] Jones DL, Nguyen C, Finlay RD (2009). Carbon flow in the rhizosphere: carbon trading at the soil–root interface. Plant Soil.

[12] Jones DL, Hodge A, Kuzyakov Y (2004). Plant and mycorrhizal regulation of rhizodeposition. New Phytol..

[13] Nguyen C (2003). Rhizodeposition of organic C by plants: mechanisms and controls. Agronomie.

[14] Sokol NW, Sanderman J, Bradford MA (2019). Pathways of mineral-associated soil organic matter formation: Integrating the role of plant carbon source, chemistry, and point of entry. Glob. Change Biol..

[15] Haddix ML, Paul EA, Cotrufo MF (2016). Dual, differential isotope labeling shows the preferential movement of labile plant constituents into mineral-bonded soil organic matter. Glob. Change Biol..

[16] Cotrufo MF (2015). Formation of soil organic matter via biochemical and physical pathways of litter mass loss. Nat. Geosci..

[17] Sokol NW, Bradford MA (2019). Microbial formation of stable soil carbon is more efficient from belowground than aboveground input. Nat. Geosci..

[18] Lavallee JM, Soong JL, Cotrufo MF (2020). Conceptualizing soil organic matter into particulate and mineral-associated forms to address global change in the 21st century. Glob. Change Biol..

[19] Liang C, Amelung W, Lehmann J, Kästner M (2019). Quantitative assessment of microbial necromass contribution to soil organic matter. Glob. Change Biol..

[20] Cotrufo MF, Wallenstein MD, Boot CM, Denef K, Paul E (2013). The Microbial Efficiency-Matrix Stabilization (MEMS) framework integrates plant litter decomposition with soil organic matter stabilization: Do labile plant inputs form stable soil organic matter?. Glob. Change Biol..

[21] Liang C, Schimel JP, Jastrow JD (2017). The importance of anabolism in microbial control over soil carbon storage. Nat. Microbiol..

[22] Meyer N (2018). Nitrogen and phosphorus supply controls soil organic carbon mineralization in tropical topsoil and subsoil. Soil Biol. Biochem..

[23] Heitkötter J, Heinze S, Marschner B (2017). Relevance of substrate quality and nutrients for microbial C-turnover in top- and subsoil of a Dystric Cambisol. Geoderma.

[24] Peixoto, L., Elsgaard, L., Rasmussen, J. & Olesen, J. E. Nitrogen and phosphorus co‐limit mineralization of labile carbon in deep subsoil. *Eur. J. Soil Sci.* (2021).

[25] Liang Z, Olesen JE, Jensen JL, Elsgaard L (2019). Nutrient availability affects carbon turnover and microbial physiology differently in topsoil and subsoil under a temperate grassland. Geoderma.

[26] Peixoto, L. *et al.* Decreased rhizodeposition, but increased microbial carbon stabilization with soil depth down to 3.6 m. *Soil Biol. Biochem.* **150**, 108008 (2020).

[27] Miltner A, Kindler R, Knicker H, Richnow H-H, Kästner M (2009). Fate of microbial biomass-derived amino acids in soil and their contribution to soil organic matter. Org. Geochem..

[28] Xia J, Wan S (2008). Global response patterns of terrestrial plant species to nitrogen addition. New Phytol..

[29] Gough L, Osenberg CW, Gross KL, Collins SL (2000). Fertilization effects on species density and primary productivity in herbaceous plant communities. Oikos.

[30] Hu T (2018). Root biomass in cereals, catch crops and weeds can be reliably estimated without considering aboveground biomass. Agr. Ecosyst. Environ..

[31] Pausch J, Kuzyakov Y (2018). Carbon input by roots into the soil: Quantification of rhizodeposition from root to ecosystem scale. Glob Chang Biol.

[32] Lu M (2011). Minor stimulation of soil carbon storage by nitrogen addition: A meta-analysis. Agr. Ecosyst. Environ..

[33] Liu L, Greaver TL (2010). A global perspective on belowground carbon dynamics under nitrogen enrichment. Ecol. Lett..

[34] Riggs CE, Hobbie SE, Bach EM, Hofmockel KS, Kazanski CE (2015). Nitrogen addition changes grassland soil organic matter decomposition. Biogeochemistry.

[35] Zang H, Wang J, Kuzyakov Y (2016). N fertilization decreases soil organic matter decomposition in the rhizosphere. Appl. Soil. Ecol..

[36] Zang H, Blagodatskaya E, Wang J, Xu X, Kuzyakov Y (2017). Nitrogen fertilization increases rhizodeposit incorporation into microbial biomass and reduces soil organic matter losses. Biol. Fertil. Soils.

[37] Schmidt R, Ulanova D, Wick LY, Bode HB, Garbeva P (2019). Microbe-driven chemical ecology: past, present and future. ISME J..

[38] Hafner S, Kuzyakov Y (2016). Carbon input and partitioning in subsoil by chicory and alfalfa. Plant Soil.

[39] Enggrob KL, Larsen T, Peixoto L, Rasmussen J (2020). Gram-positive bacteria control the rapid anabolism of protein-sized soil organic nitrogen compounds questioning the present paradigm. Sci. Rep..

[40] Miltner A, Bombach P, Schmidt-Brücken B, Kästner M (2012). SOM genesis: microbial biomass as a significant source. Biogeochemistry.

[41] Mortensen, E. Ø., De Notaris, C., Peixoto, L., Olesen, J. E. & Rasmussen, J. Short-term cover crop carbon inputs to soil as affected by long-term cropping system management and soil fertility. *Agric. Ecosyst. Environ.* **311**, 107339 (2021).

[42] Kuzyakov, Y. & Domanski, G. *Carbon Input by Plants into the Soil. Review*. Vol. 163 (2000).

[43] Rasmussen J, Gylfadóttir T, Dhalama NR, De Notaris C, Kätterer T (2019). Temporal fate of 15N and 14C leaf-fed to red and white clover in pure stand or mixture with grass–Implications for estimation of legume derived N in soil and companion species. Soil Biol. Biochem..

[44] Frostegård Å, Tunlid A, Bååth E (1991). Microbial biomass measured as total lipid phosphate in soils of different organic content. J. Microbiol. Methods.

[45] Gunina A, Dippold MA, Glaser B, Kuzyakov Y (2014). Fate of low molecular weight organic substances in an arable soil: from microbial uptake to utilisation and stabilisation. Soil Biol. Biochem..

[46] Bligh EG, Dyer WJ (1959). A rapid method of total lipid extraction and purification. Can. J. Biochem. Physiol..

[47] Zhang X, Amelung W (1996). Gas chromatographic determination of muramic acid, glucosamine, mannosamine, and galactosamine in soils. Soil Biol. Biochem..

[48] Enggrob KL, Larsen T, Larsen M, Elsgaard L, Rasmussen J (2019). The influence of hydrolysis and derivatization on the determination of amino acid content and isotopic ratios in dual-labeled (13C, 15N) white clover. Rapid Commun. Mass Spectrom..

[49] Glaser B, Gross S (2005). Compound-specific δ13C analysis of individual amino sugars—a tool to quantify timing and amount of soil microbial residue stabilization. Rapid Commun. Mass Spectrom..

[50] Dippold MA, Boesel S, Gunina A, Kuzyakov Y, Glaser B (2014). Improved delta(13)C analysis of amino sugars in soil by ion chromatography-oxidation-isotope ratio mass spectrometry. Rapid Commun. Mass Spectrom..

[51] Kušlienė G, Rasmussen J, Kuzyakov Y, Eriksen J (2014). Medium-term response of microbial community to rhizodeposits of white clover and ryegrass and tracing of active processes induced by 13C and 15N labelled exudates. Soil Biol. Biochem..

[52] Amelung W, Miltner A, Zhang X, Zech W (2001). Fate of microbial residues during litter decomposition as affected by minerals. Soil Sci..

[53] Joergensen RG (2018). Amino sugars as specific indices for fungal and bacterial residues in soil. Biol. Fertil. Soils.

[54] Kindler R, Miltner A, Thullner M, Richnow H-H, Kästner M (2009). Fate of bacterial biomass derived fatty acids in soil and their contribution to soil organic matter. Org. Geochem..

[55] Garcia-Lemos AM (2020). Under the christmas tree: Belowground bacterial associations with Abies Nordmanniana across production systems and plant development. Front. Microbiol..

[56] Callahan BJ (2016). DADA2: High-resolution sample inference from Illumina amplicon data. Nat. Methods.

[57] Martin M (2011). Cutadapt removes adapter sequences from high-throughput sequencing reads. EMBnet. J..

[58] Wang Q, Garrity GM, Tiedje JM, Cole JR (2007). Naïve Bayesian classifier for rapid assignment of rRNA sequences into the new bacterial taxonomy. Appl. Environ. Microbiol..

[59] Quast C (2012). The SILVA ribosomal RNA gene database project: Improved data processing and web-based tools. Nucleic Acids Res..

[60] Nilsson RH (2018). The UNITE database for molecular identification of fungi: Handling dark taxa and parallel taxonomic classifications. Nucleic Acids Res..

[61] McMurdie, P. J. & Holmes, S. phyloseq: an R package for reproducible interactive analysis and graphics of microbiome census data. *PloS one* **8**, e61217 (2013). 10.1371/journal.pone.0061217 PMC3632530 23630581

[62] Conover WJ, Johnson ME, Johnson MM (1981). A comparative study of tests for homogeneity of variances, with applications to the outer continental shelf bidding data. Technometrics.

[63] Royston JP (1982). An extension of Shapiro and Wilk's W test for normality to large samples. J. R Stat. Soc. Ser. C Appl. Stat..

[64] Team, R. C. R: A language and environment for statistical computing. *R Foundation for Statistical Computing, Vienna, Austria. *URL https://www.R-project.org/. (2020).

