# Supplementary material for: Deep-rooted perennial crops differ in capacity to stabilize C inputs in deep soil layers
Source: Sci Rep. 2022 Apr 8;12:5952. doi: 10.1038/s41598-022-09737-1 (PMC8993804; doi:10.1038/s41598-022-09737-1)
Supplement: Supplementary file 1 — Supplementary Information. [file 41598_2022_9737_MOESM1_ESM.pdf]

Supplementary Information for

Deep-rooted perennial crops differ in capacity to stabilize C inputs in deep soil layers

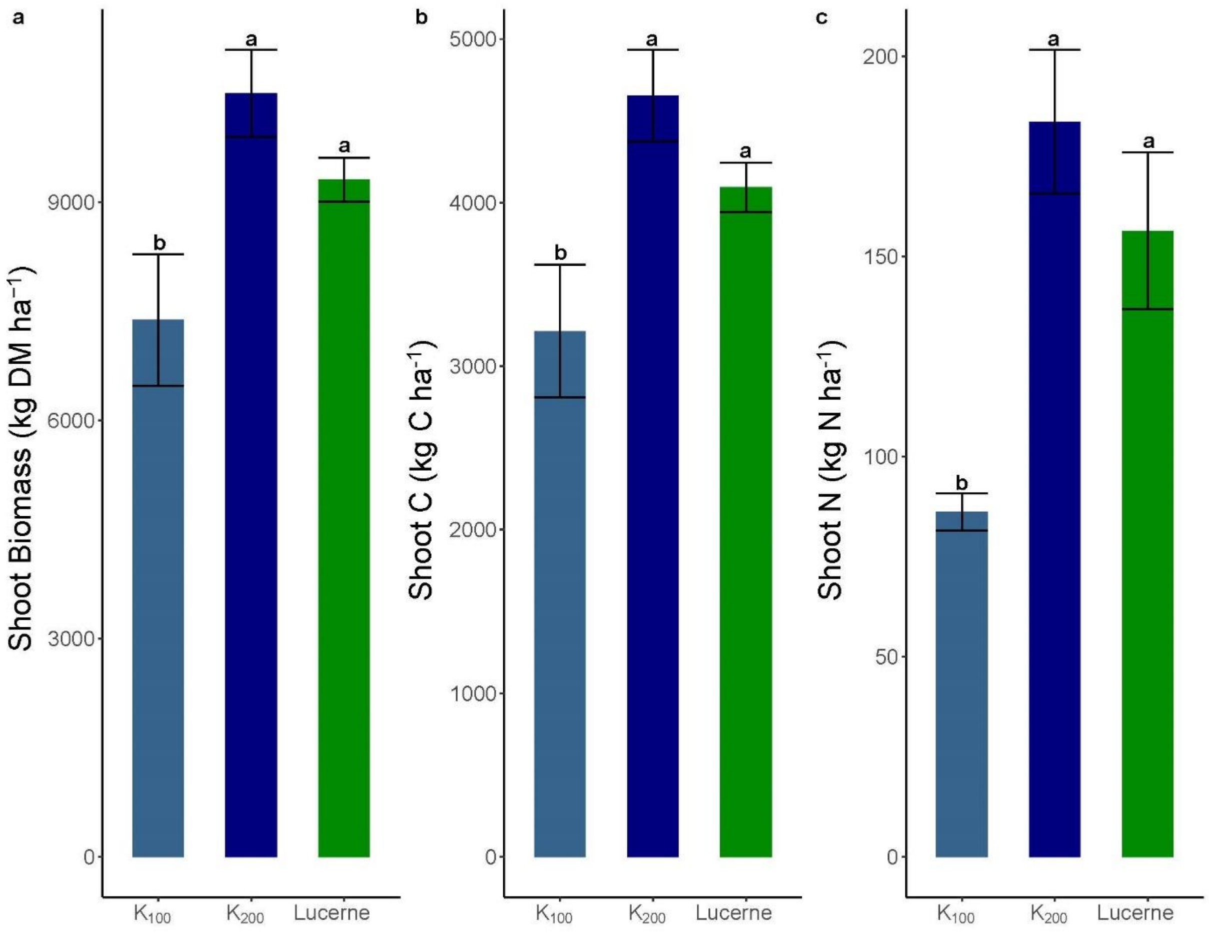

Fig. S1. Shoot biomass, carbon and nitrogen between kernza fertilized at 100 kg N ha^{-1} (K_{100}) and 200 kg N ha^{-1} (K_{200}) and lucerne. Shoot biomass (**a**), shoot carbon (C) (**b**), and shoot nitrogen (N) (**c**). Statistically significant differences ($p < 0.05$) between K_{100}, K_{200}, and lucerne in shoot biomass, shoot C, and shoot N are indicated by different letters. Error bars represent the standard error of the mean.

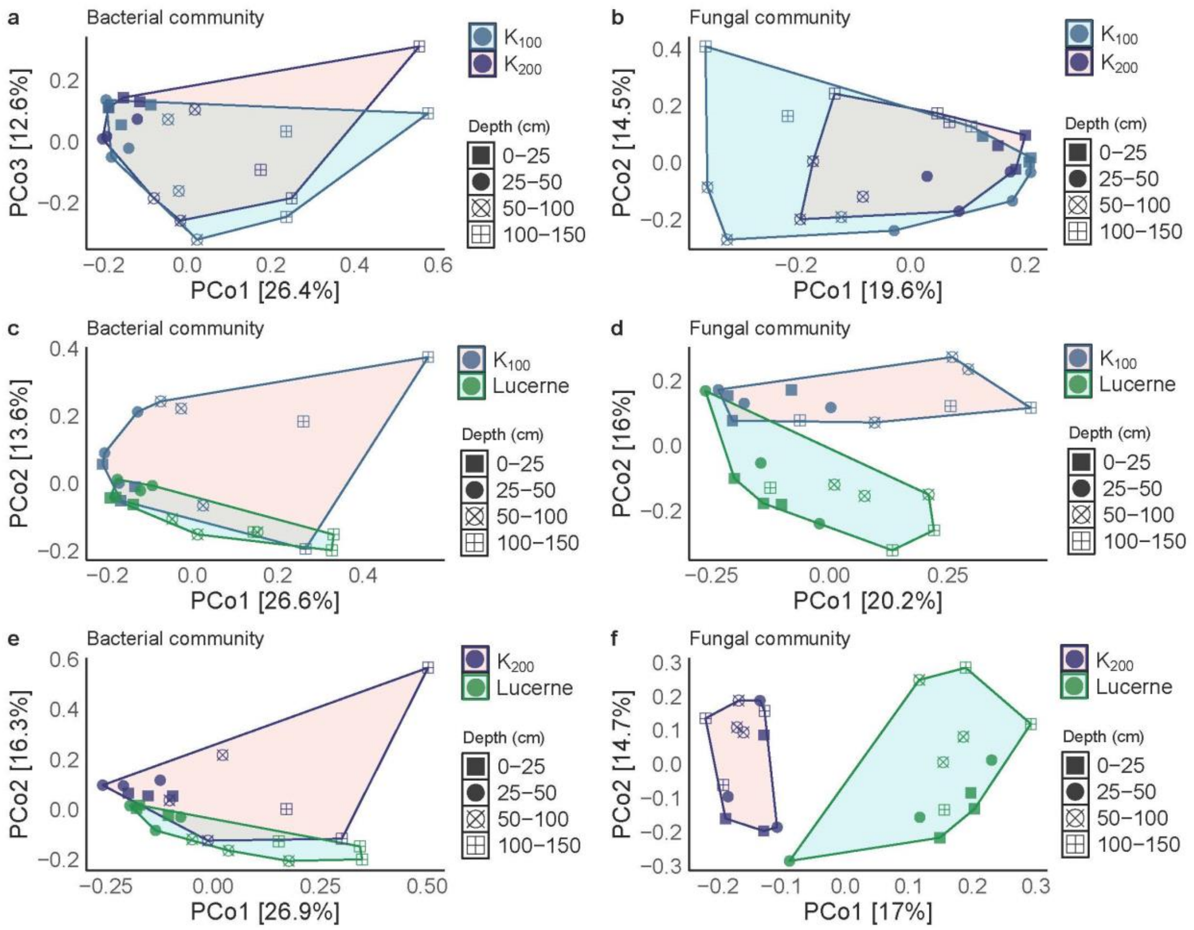

Fig. S2. PCoA ordinations of Bacterial and fungal communities using Bray-Curtis dissimilarity matrices across four depth intervals: 0-25 cm, 25-50 cm, 50-100 cm, and 100-150 cm. Bacterial community between kernza fertilized at 100 kg N ha^{-1} (K_{100}) and 200 kg N ha^{-1} (K_{200}) (p = 0.905; R^2 = 0.02) (**a**), fungal community between kernza fertilized at 100 kg N ha^{-1} (K_{100}) and 200 kg N ha^{-1} (K_{200}) (p = 0.154; R^2 = 0.05) (**b**), bacterial community between K_{100} and lucerne (p = 0.024; R^2 = 0.06) (**c**), fungal community between K_{100} and lucerne (p =0.001; R^2 = 0.124) (**d**), bacterial community between K_{200} and lucerne (p = 0.016; R^2 = 0.06) (**e**), and fungal community between K_{200} and lucerne (p = 0.001; R^2 =0.147) (**f**). Probability values are derived from the permutational multivariate analysis of variance (PERMANOVA) using the Bray-Curtis dissimilarity matrix.

Table S1. Measured explanatory variables across four depth intervals: 0-25, 25-50, 50-100, and 100-150 cm for kernza fertilized at 100 kg N ha^{-1} (K_{100}) and 200 kg N ha^{-1} (K_{200}). Different letters indicate statistically significant differences ($p < 0.05$) between each measured variable between K_{100} and K_{200} for each depth (upper case letters) and among the depths for both K_{100} and K_{200} (lower case letters). The error represents the standard error of the mean.

Measured variables	Depth (cm)	K_{100}	K_{200}
**Soil organic carbon (SOC)**	0-25	12.28 ± 2.50 **a(A)**	12.63 ± 2.44 **a(A)**
g C kg^{-1} dw soil	25-50	7.18 ± 2.12 **a(A)**	8.74 ± 2.37 **a(A)**
	50-100	2.96 ± 0.19 **b(A)**	4.07 ± 1.39 **b(A)**
	100-150	1.87 ± 0.39 **b(A)**	2.32 ± 0.07 **b(A)**
**Soil total nitrogen (TN)**	0-25	1.50 ± 0.15 **a(A)**	1.35 ± 0.26 **a(A)**
g N kg^{-1} dw soil	25-50	0.93 ± 0.17 **a(A)**	1.03 ± 0.27 **ab(A)**
	50-100	0.47 ± 0.06 **b(A)**	0.52 ± 0.15 **bc(A)**
	100-150	0.31 ± 0.10 **b(A)**	0.23 ± 0.05 **c(A)**
**Recovered roots**	0-25	6.08 ± 2.28 **a(A)**	6.78 ± 3.83 **a(A)**
g of roots kg^{-1} dw soil	25-50	1.43 ± 0.97 **b(A)**	1.30 ± 0.89 **b(A)**
	50-100	0.58 ± 0.15 **b(A)**	0.49 ± 0.11 **b(A)**
	100-150	0.12 ± 0.08 **c(A)**	0.09 ± 0.02 **c(A)**
**Root TN**	0-25	44.70 ± 19.61 **a(A)**	56.65 ± 33.02 **a(A)**
mg N kg^{-1} dw soil	25-50	8.40 ± 5.60 **b(A)**	5.82 ± 3.74 **b(A)**
	50-100	2.42 ± 0.85 **b(A)**	2.88 ± 0.71 **b(A)**
	100-150	0.58 ± 0.32 **c(A)**	0.56 ± 0.04 **c(A)**
**Root C:N ratio**	0-25	54.59 ± 6.94 **b(A)**	42.96 ± 4.76 **b(A)**
	25-50	60.73 ± 3.25 **b(B)**	70.55 ± 1.40 **ac(A)**
	50-100	96.11 ± 15.27 **a(A)**	86.48 ± 6.96 **a(A)**
	100-150	68.85 ± 8.32 **ab(A)**	54.79 ± 8.93 **bc(A)**
**Net rhizodeposited N**	0-25	*cannot solely be attributed to rhizodeposition	
g N kg^{-1} dw soil	25-50	*cannot solely be attributed to rhizodeposition	
	50-100	0.01 ± 0.006 **b(A)**	0.02 ± 0.004 **b(A)**
	100-150	0.001 ± 0.0004 **c(A)**	0.0002 ± 0.0001 **c(A)**
**Phospholipid fatty acids (PLFA)**	0-25	35.67 ± 11.81 **a(A)**	40.03 ± 10.20 **a(A)**
µg PLFA g^{-1} dw soil	25-50	7.72 ± 2.80 **b(A)**	16.34 ± 4.03 **a(A)**
	50-100	4.44 ± 2.37 **bc(A)**	2.47 ± 1.40 **b(A)**
	100-150	1.37 ± 1.13 **c(A)**	0.32 ± 0.31 **c(A)**
**^{13}C PLFA**	0-25	0.01 ± 0.006 **a(A)**	0.005 ± 0.002 **a(A)**
µg ^{13}C PLFA g^{-1} dw soil	25-50	0.0007 ± 0.0003 **b(A)**	0.0007 ± 0.0003 **b(A)**
	50-100	0.0004 ± 0.0004 **b(A)**	0.0001 ± 0.00005 **c(A)**
**Amino sugars**	0-25	487.08 ± 88.34 **a(A)**	368.33 ± 77.39 **a(A)**

µg AS g^{-1} dw soil	25-50	411.69 ± 146.51 **a(A)**	315.39 ± 84.05 **a(A)**
	50-100	158.81 ± 23.34 **b(A)**	137.25 ± 15.42 **b(A)**
	100-150	104.91 ± 15.64 **b(A)**	41.82 ± 19.50 **c(B)**
**^{13}C amino sugars**	0-25	0.02 ± 0.004 **a(A)**	0.009 ± 0.002 **a(A)**
µg ^{13}C AS g^{-1} dw soil	25-50	0.02 ± 0.009 **a(A)**	0.01 ± 0.004 **a(A)**
	50-100	0.01 ± 0.004 **a(A)**	0.01 ± 0.001 **a(A)**
**Amino acids (soil)**	0-25	1.23 ± 0.27 **a(A)**	0.68 ± 0.42 **a(A)**
mg AA g^{-1} dw soil	25-50	0.67 ± 0.21 **b(A)**	0.86 ± 0.47 **a(A)**
	50-100	0.19 ± 0.02 **c(A)**	0.32 ± 0.14 **a(A)**
**Amino acids (root)**	0-25	11.95 ± 0.63 **a(A)**	14.50 ± 0.91 **a(A)**
mg AA g^{-1} dw root	25-50	9.85 ± 0.76 **ab**	No data
	50-100	7.18 ± 2.30 **b(A)**	7.42 ± 0.89 **b(A)**
**^{13}C Amino acids (soil)**	0-25	0.06 ± 0.01 **a(A)**	0.03 ± 0.01 **a(A)**
mg ^{13}C AA g^{-1} dw soil	25-50	0.03 ± 0.009 **b(A)**	0.03 ± 0.02 **a(A)**
	50-100	0.01 ± 0.0009 **c(A)**	0.01 ± 0.007 **a(A)**
**^{13}C Amino acids (root)**	0-25	1.49 ± 0.39 **a(A)**	3.14 ± 1.32 **a(A)**
mg ^{13}C AA g^{-1} dw root	25-50	1.24 ± 0.12 **a**	No data
	50-100	0.70 ± 0.49 **a(A)**	0.73 ± 0.17 **b(A)**
**Bacterial abundance**	0-25	6.54 ± 0.09 **b(A)**	7.03 ± 0.23 **a(A)**
Log 16S rRNA gene copies g^{-1} dw soil	25-50	7.45 ± 0.04 **a(A)**	7.26 ± 0.11 **a(B)**
	50-100	7.35 ± 0.0001 **a(A)**	7.26 ± 0.14 **a(A)**
	100-150	6.90 ± 0.21 **b(A)**	7.07 ± 0.12 **a(B)**
**Fungal abundance**	0-25	8.61 ± 0.02 **a(A)**	8.59 ± 0.07 **a(A)**
Log ITS copies g^{-1} dw soil	25-50	8.23 ± 0.08 **ab(A)**	8.20 ± 0.03 **b(A)**
	50-100	7.73 ± 0.04 **b(A)**	7.74 ± 0.15 **c(A)**
	100-150	7.12 ± 0.38 **c(A)**	7.48 ± 0.15 **c(A)**
**Nitrite oxidative gene (*nirS*)**	0-25	0.73 ± 0.11 **a(A)**	0.36 ± 0.13 **a(A)**
ratio of nirS:16S rRNA gene copies	25-50	0.08 ± 0.02 **c(A)**	0.09 ± 0.02 **b(A)**
	50-100	0.06 ± 0.01 **c(A)**	0.07 ± 0.01 **b(A)**
	100-150	0.31 ± 0.13 **b(A)**	0.40 ± 0.14 **a(A)**
**Ammonia oxidative gene (*amoA*)**	0-25	1.05 ± 0.13 **a(A)**	0.50 ± 0.29 **a(A)**
ratio of amoA:16S rRNA gene copies	25-50	0.04 ± 0.02 **b(A)**	0.04 ± 0.02 **b(A)**
	50-100	0.003 ± 0.0005 **c(A)**	0.006 ± 0.002 **c(A)**
	100-150	0.006 ± 0.005 **c(A)**	0.01 ± 0.006 **bc(A)**
**Nitrogenase gene (*nifH*)**	0-25	0.54 ± 0.09 **a (A)**	0.18 ± 0.07 **ab (B)**
ratio of nifH:16S rRNA gene copies	25-50	0.06 ± 0.01 **c(A)**	0.08 ± 0.02 **b(A)**
	50-100	0.06 ± 0.02 **c(A)**	0.07 ± 0.01 **b(A)**

	100-150	0.20 ± 0.04 **b(A)**	0.34 ± 0.07 **a(A)**
**Nitrite oxidative gene (*nirK*)**	0-25	1.04 ± 0.07 **a(A)**	0.38 ± 0.11 **a(B)**
ratio of nirK:16S rRNA gene copies	25-50	0.13 ± 0.006 **b(A)**	0.13 ± 0.02 **b(A)**
	50-100	0.08 ± 0.004 **c(A)**	0.10 ± 0.01 **b(A)**
	100-150	0.10 ± 0.01 **c(A)**	0.13 ± 0.004 **b(A)**
**Nitrous oxide reductase gene (*nosZ*)**	0-25	2.80 ± 0.57 **a(A)**	0.87 ± 0.42 **a(B)**
ratio of nosZ:16S rRNA gene copies	25-50	0.10 ± 0.02 **b(A)**	0.14 ± 0.04 **b(A)**
	50-100	0.04 ± 0.005 **c(A)**	0.05 ± 0.001 **c(A)**
	100-150	0.05 ± 0.008 **c(A)**	0.04 ± 0.01 **c(A)**

Table S2. Measured explanatory variables across four depth intervals: 0-25, 25-50, 50-100, and 100-150 cm for kernza and lucerne. Different letters indicate statistically significant differences ($p < 0.05$) for each measured variable between lucerne and kernza for each depth (upper case letters) and among the depths for each plant species (lower case letters). The error represents the standard error of the mean. When significant differences between K_{100} and K_{200} were detected, lucerne was compared to both K_{100} and K_{200} as indicated by **(K_{100})** and **(K_{200})**.

**Measured variables**	**Depth (cm)**	**Kernza**	**Lucerne**
**Soil organic carbon (SOC)**	0-25	12.46 ± 1.76 **a(A)**	13.83 ± 1.39 **a(A)**
g C kg^{-1} dw soil	25-50	7.96 ± 1.58 **a(A)**	9.76 ± 2.09 **a(A)**
	50-100	3.51 ± 0.78 **b(A)**	5.60 ± 1.86 **b(A)**
	100-150	2.10 ± 0.17 **b(A)**	1.87 ± 0.29 **c(A)**
**Soil total nitrogen (TN)**	0-25	1.43 ± 0.20 **a(A)**	1.50 ± 0.15 **a(A)**
g N kg^{-1} dw soil	25-50	0.98 ± 0.18 **a(A)**	1.07 ± 0.22 **ab(A)**
	50-100	0.49 ± 0.09 **b(A)**	0.60 ± 0.20 **b(A)**
	100-150	0.27 ± 0.08 **c(A)**	0.17 ± 0.03 **c(A)**
**Recovered roots**	0-25	6.43 ± 3.05 **a(B)**	8.20 ± 0.96 **a(A)**
g of roots kg^{-1} dw soil	25-50	0.96 ± 0.46 **b(A)**	1.03 ± 0.45 **b(A)**
	50-100	0.94 ± 0.47 **b(A)**	0.31 ± 0.11 **c(A)**
	100-150	0.10 ± 0.04 **c(A)**	0.08 ± 0.02 **d(A)**
**Root N**	0-25	50.67 ± 26.25 **a(A)**	107.58 ± 31.14 **a(A)**
mg N kg^{-1} dw soil	25-50	7.11 ± 2.90 **b(A)**	12.70 ± 5.02 **b(A)**
	50-100	2.65 ± 0.35 **b(A)**	4.94 ± 1.91 **c(A)**
	100-150	0.57 ± 0.17 **c(B)**	1.61 ± 0.32 **d(A)**
**Root C:N ratio**	0-25	48.78 ± 4.73 **b(B)**	26.89 ± 0.45 **ab(A)**
	25-50 **(K_{100})**	60.73 ± 3.25 **b(B)**	30.18 ± 2.14 **a(A)**
	25-50 **(K_{200})**	70.55 ± 1.40 **(B)**	30.18 ± 2.14 **(A)**
	50-100	91.29 ± 7.86 **a(B)**	25.85 ± 0.39 **b(A)**
	100-150	61.82 ± 7.00 **b(B)**	20.44 ± 0.89 **c(A)**
**Net rhizodeposited N**	0-25	*cannot solely be attributed to rhizodeposition	
g N kg^{-1} dw soil	25-50	*cannot solely be attributed to rhizodeposition	
	50-100	0.01 ± 0.003 **b(B)**	0.04 ± 0.01 **b(A)**
	100-150	0.0004 ± 0.0003 **c(A)**	0.0001 ± 0.0001 **c(A)**

**Phospholipid fatty acids (PLFA)**	0-25	38.28 ± 7.33 **a(B)**	70.50 ± 13.04 **a(A)**
µg PLFA g^{-1} dw soil	25-50	12.42 ± 2.77 **b(A)**	19.47 ± 5.83 **b(A)**
	50-100	3.46 ± 1.33 **c(B)**	7.26 ± 1.50 **b(A)**
	100-150	0.95 ± 0.68 **d(A)**	0.92 ± 0.53 **c(A)**
**^{13}C PLFA**	0-25	0.008 ± 0.004 **a(A)**	0.010 ± 0.005 **a(A)**
µg ^{13}C PLFA g^{-1} dw soil	25-50	0.001 ± 0.0003 **b(A)**	0.001 ± 0.0005 **b(A)**
	50-100	0.0004 ± 0.0002 **b(A)**	0.0003 ± 0.0001 **c(A)**
**Amino sugars**	0-25	427.71 ± 49.96 **a(A)**	532.30 ± 28.72 **a(A)**
µg AS g^{-1} dw soil	25-50	363.54 ± 112.83 **a(A)**	412.70 ± 106.52 **a(A)**
	50-100	148.03 ± 11.61 **b(A)**	105.31 ± 24.06 **b(A)**
	100-150 **(K_{100})**	104.91 ± 15.64 **b(A)**	32.06 ± 3.27 **c(B)**
	100-150 **(K_{200})**	41.82 ± 19.50 **(A)**	32.06 ± 3.27 **(A)**
**^{13}C amino sugars**	0-25	0.01 ± 0.002 **a(B)**	0.04 ± 0.01 **a(A)**
µg ^{13}C AS g^{-1} dw soil	25-50	0.02 ± 0.01 **a(A)**	0.02 ± 0.002 **b(A)**
	50-100	0.01 ± 0.002 **a(A)**	0.01 ± 0.004 **b(A)**
**Amino acids (soil)**	0-25	0.95 ± 0.31 **a(A)**	1.32 ± 0.39 **a(A)**
mg AA g^{-1} dw soil	25-50	0.76 ± 0.34 **a(A)**	1.00 ± 0.31 **a(A)**
	50-100	0.26 ± 0.08 **b(B)**	0.54 ± 0.13 **b(A)**
**Amino acids (root)**	0-25	12.66 ± 0.92 **a(B)**	26.40 ± 2.07 **a(A)**
mg AA g^{-1} dw root	25-50 **(K_{100})**	9.85 ± 0.76 **b(B)**	20.33 ± 0.63 **a(A)**
	25-50 **(K_{200})**	No data	
	50-100	7.04 ± 1.00 **c(B)**	26.31 ± 1.54 **a(A)**
**^{13}C Amino acids (soil)**	0-25	0.04 ± 0.01 **a(A)**	0.03 ± 0.01 **a(A)**
mg ^{13}C AA g^{-1} dw soil	25-50	0.03 ± 0.01 **ab(A)**	0.03 ± 0.01 **a(A)**
	50-100	0.01 ± 0.004 **b(A)**	0.01 ± 0.004 **a(A)**
**^{13}C Amino acids (root)**	0-25	1.42 ± 0.32 **a(B)**	3.57 ± 0.59 **ab(A)**
mg ^{13}C AA g^{-1} dw root	25-50 **(K_{100})**	1.24 ± 0.12 **a(B)**	2.54 ± 0.86 **b(A)**
	25-50 **(K_{200})**	No data	
	50-100	0.67 ± 0.21 **b(B)**	4.43 ± 0.56 **a(A)**
**Bacterial abundance**	0-25	6.87 ± 0.18 **b(A)**	6.54 ± 0.12 **b(A)**
Log 16S rRNA gene copies g^{-1} dw soil	25-50	7.38 ± 0.02 **a(A)**	7.28 ± 0.02 **a(B)**
	50-100	7.32 ± 0.06 **a(A)**	7.36 ± 0.01 **a(A)**
	100-150	7.03 ± 0.09 **b(A)**	6.73 ± 0.05 **b(B)**
**Fungal abundance**	0-25	8.60 ± 0.04 **a(A)**	8.53 ± 0.04 **a(A)**
Log ITS copies g^{-1} dw soil	25-50	8.22 ± 0.03 **b(A)**	8.23 ± 0.08 **b(A)**
	50-100	7.75 ± 0.07 **c(A)**	7.72 ± 0.05 **c(A)**
	100-150	7.37 ± 0.23 **d(A)**	7.13 ± 0.12 **d(A)**
**Nitrite oxidative gene (*nirS*)**	0-25 **(K_{100})**	0.73 ± 0.11 **a(A)**	0.68 ± 0.13 **a(A)**
ratio of nirS:16S rRNA gene copies	0-25 **(K_{200})**	0.36 ± 0.13 **(A)**	0.68 ± 0.13 **(A)**
	25-50	0.09 ± 0.02 **c(A)**	0.14 ± 0.01 **c(A)**
	50-100	0.07 ± 0.01 **c(B)**	0.22 ± 0.01 **b(A)**
	100-150	0.36 ± 0.13 **b(A)**	0.34 ± 0.08 **b(A)**
**Ammonia oxidative gene (*amoA*)**	0-25 **(K_{100})**	1.05 ± 0.13 **a(A)**	1.53 ± 0.52 **a(A)**
ratio of amoA:16S rRNA gene copies	0-25 **(K_{200})**	0.50 ± 0.29 **(A)**	1.53 ± 0.52 **(A)**

	25-50	0.02 ± 0.01 **b(B)**	0.06 ± 0.02 **b(A)**
	50-100	0.002 ± 0.001 **c(B)**	0.01 ± 0.001 **c(A)**
	100-150	0.002 ± 0.001 **c(B)**	0.01 ± 0.003 **c(A)**
**Nitrogenase gene (*nifH*)**	0-25 **(K_{100})**	0.54 ± 0.09 **a(A)**	0.45 ± 0.15 **a(A)**
ratio of nifH:16S rRNA gene copies	0-25 **(K_{200})**	0.18 ± 0.07 **(A)**	0.45 ± 0.15 **(A)**
	25-50	0.07 ± 0.01 **c(A)**	0.09 ± 0.01 **c(A)**
	50-100	0.07 ± 0.02 **c(B)**	0.19 ± 0.03 **b(A)**
	100-150	0.27 ± 0.05 **b(B)**	0.45 ± 0.06 **a(A)**
**Nitrite oxidative gene (*nirK*)**	0-25 **(K_{100})**	1.04 ± 0.07 **a(A)**	1.21 ± 0.47 **a(A)**
ratio of nirK:16S rRNA gene copies	0-25 **(K_{200})**	0.38 ± 0.11 **(A)**	1.21 ± 0.47 **(A)**
	25-50	0.13 ± 0.01 **b(A)**	0.16 ± 0.01 **b(A)**
	50-100	0.08 ± 0.002 **c(A)**	0.10 ± 0.01 **b(A)**
	100-150	0.10 ± 0.01 **c(B)**	0.14 ± 0.01 **b(A)**
**Nitrous oxide reductase gene (*nosZ*)**	0-25 **(K_{100})**	2.80 ± 0.57 **a(A)**	3.34 ± 1.58 **a(A)**
ratio of nosZ:16S rRNA gene copies	0-25 **(K_{200})**	0.87 ± 0.42 **(A)**	3.34 ± 1.58 **(A)**
	25-50	0.12 ± 0.02 **b(A)**	0.16 ± 0.02 **b(A)**
	50-100	0.04 ± 0.002 **c(B)**	0.06 ± 0.004 **c(A)**
	100-150	0.05 ± 0.01 **c(A)**	0.06 ± 0.01 **c(A)**

Table S3. The relative abundance of unique bacterial and fungal amplicon sequencing variants (ASVs) for kernza fertilized at 100 kg N ha^{-1} (K_{100}) and 200 kg N ha^{-1} (K_{200}) and lucerne at four depth intervals (0-150 cm).

	**Bacterial ASVs**			**Fungal ASVs**		
**Depth (cm)**	**K_{100}**	**K_{200}**	**Lucerne**	**K_{100}**	**K_{200}**	**Lucerne**
0-25	3.9%	3.8%	4.9%	2.0%	1.9%	2.4%
25-50	5.0%	6.0%	5.4%	2.2%	4.2%	1.9%
50- 100	7.5%	7.4%	6.7%	3.4%	4.3%	2.9%
100-150	11.1%	14.4%	10.9%	6.4%	5.5%	8.5%

Table S4. Texture of the bulk soil in the four depth intervals sampled (0-150 cm).

**Depth (cm)**	**Bulk density ($g\ cm^{-3}$)**	**Clay (%)**	**Silt (%)**	**Sand (%)**	**pH ($CaCl_2$)**
0-25	1.43 ± 0.03	14.61 ± 0.21	16.12 ± 1.41	67.91 ± 1.51	7.71 ± 0.03
25-50	1.52 ± 0.03	19.73 ± 1.70	13.40 ± 1.52	66.87 ± 2.74	7.80 ± 0.06
50-100	1.74 ± 0.003	19.78 ± 0.81	15.37 ± 1.23	64.85 ± 2.01	7.75 ± 0.14
100-150	1.75 ± 0.004	19.63 ± 0.58	17.33 ± 1.17	63.04 ± 2.65	7.79 ± 0.31

Table S5. Primers used for qPCR and Illumina amplicon sequencing.

Target region	Primers	Sequences (5’ to 3’)	Annealing Temperature	Reference
16S rRNA	341F	CCT AYG GGR BGC ASC AG		(Sundberg *et al.*, 2013)
Illumina	806R	GGA CTA CNN GGG TAT CTA AT		(Sundberg *et al.*, 2013)
16S rRNA	341F	CCT AYG GGR BGC ASC AG	58°C	(Sundberg *et al.*, 2013)
qPCR	805R	GAC TAC HVG GGT ATC TAA TCC		(Herlemann *et al.*, 2011)
ITS 1[a]	ITS1	CTT GGT CAT TTA GAG GAA GTA A	55°C	(Gardes and Bruns, 1993)
	ITS2	GCT GCG TTC TTC ATC GAT GC		(White *et al.*, 1990)
nirK	nirK-F1aCu	ATC ATG GTS CTG CCG CG	58°C	(Hallin and Lindgren, 1999)
	nirK-R3Cu	GCC TCG ATC AGR TTG TGG TT		(Hallin and Lindgren 1999)
nosZ	nosZ-F	CGY TGT TCM TCG ACA GCC AG	63°C	(Kloos *et al.*, 2001)
	nosZ-1622R	CGS ACC TTS TTG CCS TYG CG		(Throbäck *et al.*, 2004)
nifH	nifHF	AAA GGY GGW ATC GGY AAR TCC ACC AC	55°C	(Rösch and Bothe, 2005)
	nifHRb	TGS GCY TTG TCY TCR CGG ATB GGC AT		(Rösch and Bothe, 2005)
nirS	nirS cd3AF	GTS AAC GTS AAG GAR ACS GG	55°C	(Throbäck *et al.* 2004)
	nirS R3cd	GAS TTC GGR TGS GTC TTG A		(Throbäck *et al.* 2004)
amoA	amoA-1Fmod	CTG GGG TTT CTA CTG GTG GTC	58°C	(Meinhardt *et al.*, 2015)
	GenAOBR-1	GCA GTG ATC ATC CAG TTG CG		(Meinhardt *et al.* 2015)

a) Primers used for qPCR and Illumina amplicon sequencing.

SI References

Gardes, M., Bruns, T.D., 1993. ITS primers with enhanced specificity for basidiomycetes-application to the identification of mycorrhizae and rusts. Molecular ecology 2, 113-118.

Hallin, S., Lindgren, P.-E., 1999. PCR detection of genes encoding nitrite reductase in denitrifying bacteria. Applied and environmental microbiology 65, 1652-1657.

Herlemann, D.P., Labrenz, M., Jürgens, K., Bertilsson, S., Waniek, J.J., Andersson, A.F., 2011. Transitions in bacterial communities along the 2000 km salinity gradient of the Baltic Sea. The ISME journal 5, 1571-1579.

Kloos, K., Mergel, A., Rösch, C., Bothe, H., 2001. Denitrification within the genus Azospirillum and other associative bacteria. Functional Plant Biology 28, 991-998.

Meinhardt, K.A., Bertagnolli, A., Pannu, M.W., Strand, S.E., Brown, S.L., Stahl, D.A., 2015. Evaluation of revised polymerase chain reaction primers for more inclusive quantification of ammonia-oxidizing archaea and bacteria. Environmental microbiology reports 7, 354-363.

Rösch, C., Bothe, H., 2005. Improved assessment of denitrifying, N2-fixing, and total-community bacteria by terminal restriction fragment length polymorphism analysis using multiple restriction enzymes. Applied and environmental microbiology 71, 2026-2035.

Sundberg, C., Al-Soud, W.A., Larsson, M., Alm, E., Yekta, S.S., Svensson, B.H., Sørensen, S.J., Karlsson, A., 2013. 454 pyrosequencing analyses of bacterial and archaeal richness in 21 full-scale biogas digesters. FEMS microbiology ecology 85, 612-626.

Throbäck, I.N., Enwall, K., Jarvis, Å., Hallin, S., 2004. Reassessing PCR primers targeting nirS, nirK and nosZ genes for community surveys of denitrifying bacteria with DGGE. FEMS microbiology ecology 49, 401-417.

White, T.J., Bruns, T., Lee, S., Taylor, J., 1990. Amplification and direct sequencing of fungal ribosomal RNA genes for phylogenetics. PCR protocols: a guide to methods and applications 18, 315-322.
